# Genomic ancestry and cancer among Latin Americans

**DOI:** 10.1007/s12094-024-03415-6

**Published:** 2024-04-06

**Authors:** Alejandro Ruíz-Patiño, Leonardo Rojas, Jairo Zuluaga, Oscar Arrieta, Luis Corrales, Claudio Martín, Sandra Franco, Luis Raez, Christian Rolfo, Natalia Sánchez, Andrés Felipe Cardona

**Affiliations:** 1 Clinical Genetics, Luis Carlos Sarmiento Angulo Cancer Treatment and Research Center (CTIC), Bogotá, Colombia; 2 Foundation for Clinical and Applied Cancer Research – FICMAC, Bogotá, Colombia; 3 GIGA/TERA Research Group, CTIC/Universidad El Bosque, Bogotá, Colombia; 4 Thoracic Oncology Unit, Luis Carlos Sarmiento Angulo Cancer Treatment and Research Center (CTIC), Bogotá, Colombia; 5 Instituto Nacional de Cancerología –INCaN, Mexico City, Mexico; 6 Thoracic Oncology Unit, Centro de Investigación y Manejo del Cáncer (CIMCA), San José, Costa Rica; 7 Thoracic Oncology Unit, Instituto Alexander Fleming, Buenos Aires, Argentina; 8 Breast Cancer Unit, Luis Carlos Sarmiento Angulo Cancer Treatment and Research Center (CTIC), Bogotá, Colombia; 9 Oncology Department, Memorial Cancer Institute (MCI), Memorial Healthcare System, Miami, FL USA; 10 Center for Thoracic Oncology, Tisch Cancer Institute, Icahn School of Medicine at Mount Sinai, New York, NY USA; 11 Institute of Research, Science and Education, Luis Carlos Sarmiento Angulo Cancer Treatment and Research Center (CTIC), Bogotá, Colombia; 12 Direction of Research and Education, Luis Carlos Sarmiento Angulo Cancer Treatment and Research Center (CTIC), Cra. 14 #169-49, Bogotá, Colombia

**Keywords:** Cancer, Genetic ancestry, Genetic epidemiology, NA (NA), Genome‐wide association study, Polygenic risk score

## Abstract

Latin American populations, characterized by intricate admixture patterns resulting from the intermingling of ancestries from European, Native American (NA) Asian, and African ancestries which result in a vast and complex genetic landscape, harboring unique combinations of novel variants. This genetic diversity not only poses challenges in traditional population genetics methods but also opens avenues for a deeper understanding of its implications in health. In cancer, the interplay between genetic ancestry, lifestyle factors, and healthcare disparities adds a layer of complexity to the varying incidence and mortality rates observed across different Latin American subpopulations. This complex interdependence has been unveiled through numerous studies, whether conducted on Latin American patients residing on the continent or abroad, revealing discernible differences in germline composition that influence divergent disease phenotypes such as higher incidence of Luminal B and Her2 breast tumors, EGFR and KRAS mutated lung adenocarcinomas in addition to an enrichment in BRCA1/2 pathogenic variants and a higher than expected prevalence of variants in colorectal cancer associated genes such as APC and MLH1. In prostate cancer novel risk variants have also been solely identified in Latin American populations. Due to the complexity of genetic divergence, inputs from each individual ancestry seem to carry independent contributions that interplay in the development of these complex disease phenotypes. By understanding these unique population characteristics, genomic ancestries hold a promising avenue for tailoring prognostic assessments and optimizing responses to oncological interventions.

## Introduction

Ancestry within populations formed through admixture showcases variations across various genetic scales, occurring in individuals and in their respective genomes [1]. Numerous methods in population genetics and their resulting analyses rely on assumptions about populations, which may no longer hold true in the context of recent admixture events. In scenarios where isolation is the prevailing model, measurements associated with genomic diversity typically possess clearly delineated theoretical predictions regarding fundamental parameters in the population’s evolutionary trajectory [2]. Nevertheless, the interconnections among these metrics can become uncertain with the presence of admixture, which introduces groupings of interconnected ancestral haplotypes. Each cluster of haplotypes might exhibit unique variations influenced by the historical origins of the original populations. Essentially, admixture disturbs both the structures of genetic linkage and the distributions of allele frequencies, elements often neglected in conventional inference methods formulated without accounting for the influence of this phenomenon [3].

Admixed genomes have not only eased the discovery of connections between genetic variants and traits but have also propelled improvements in genetic risk prediction models beyond the capabilities of associations and predictions derived solely from ancestral populations [4]. Recent improvements in methodology have enhanced the accuracy and effectiveness of local ancestry calling. This advancement enables the deduction of local ancestry patterns within the populations that prove to be admixed, providing insights into the history of their demographic, their adaptation, as well as the vast genetic underpinnings of complex traits, including cancer-related ones [5].

The genomes of Latin Americans represent a recent evolutionary development, combining haplotypes that have not coexisted together before within a shared genetic setting. Contemporary Latin American populations display genetically distinctive genomes, showcasing a blend of ancestries ranging from Europe, Native America and Sub-Saharan Africa. The distribution of these ancestral components varies notably among countries and even among subgroups within each country [6, 7] Different genetic markers, such as whole-genome sequencing (WGS), high-density single nucleotide polymorphism (SNP) datasets, microsatellites, short tandem repeats (STRs), insertions or deletions (InDels), and the ABO blood system, have been employed to understand these distinctions [8–10]. Understanding data from both ancestry and genomes has spurred the inception of population-based initiatives in Latin America (LATAM). For example, among Mexican individuals, there has been significant selection for African ancestry within the major histocompatibility complex (MHC) [11]. A study conducted in the Caribbean coincidentally revealed that female patients diagnosed with type II diabetes and obesity displayed a greater proportion of the ancestry of sub-Saharan Africa in comparison to other women without these health conditions [12]. In Colombia, a study involving 624 individuals analyzing ancestry revealed stronger correlations between disease prevalence risk, estimated by polygenic risk scores (PRS), and origin [13]. Furthermore, in the Peruvian Genome Project and the 12G/100G-MX Project [14, 15] data from the whole genomes of NA populations were utilized, emphasizing susceptibility to tuberculosis development. Moreover, “The Mexico City Prospective Study” examined high-density single nucleotide polymorphism (SNP) genotype data and conducted whole-exome/whole-genome sequencing in more than 140,000 adults from Mexico, associating the results with phenotypic traits such as obesity and diabetes [16]. Findings from the Brazilian Initiative on Precision Medicine (BIPMED) similarly presented data derived from SNP arrays and whole-exome sequencing (WES). Notably, they noted a reduction in the proportion of European ancestry and an overabundance of NA ancestry on chromosome 8p23.1. This chromosomal segment harbors genes linked to obesity, type 2 diabetes, lipid levels, and waist circumference [17]. In 2021, the JAGUAR project (Joining all: Genes, immUnity, and diveRsity) commenced with the objective of mapping immune cells throughout Latin America. This initiative strives to develop the initial high-resolution genetic atlas encompassing various ancestries, with the goal of comprehending the influence of ancestry on the immune system [18].

**Fig. 1:**
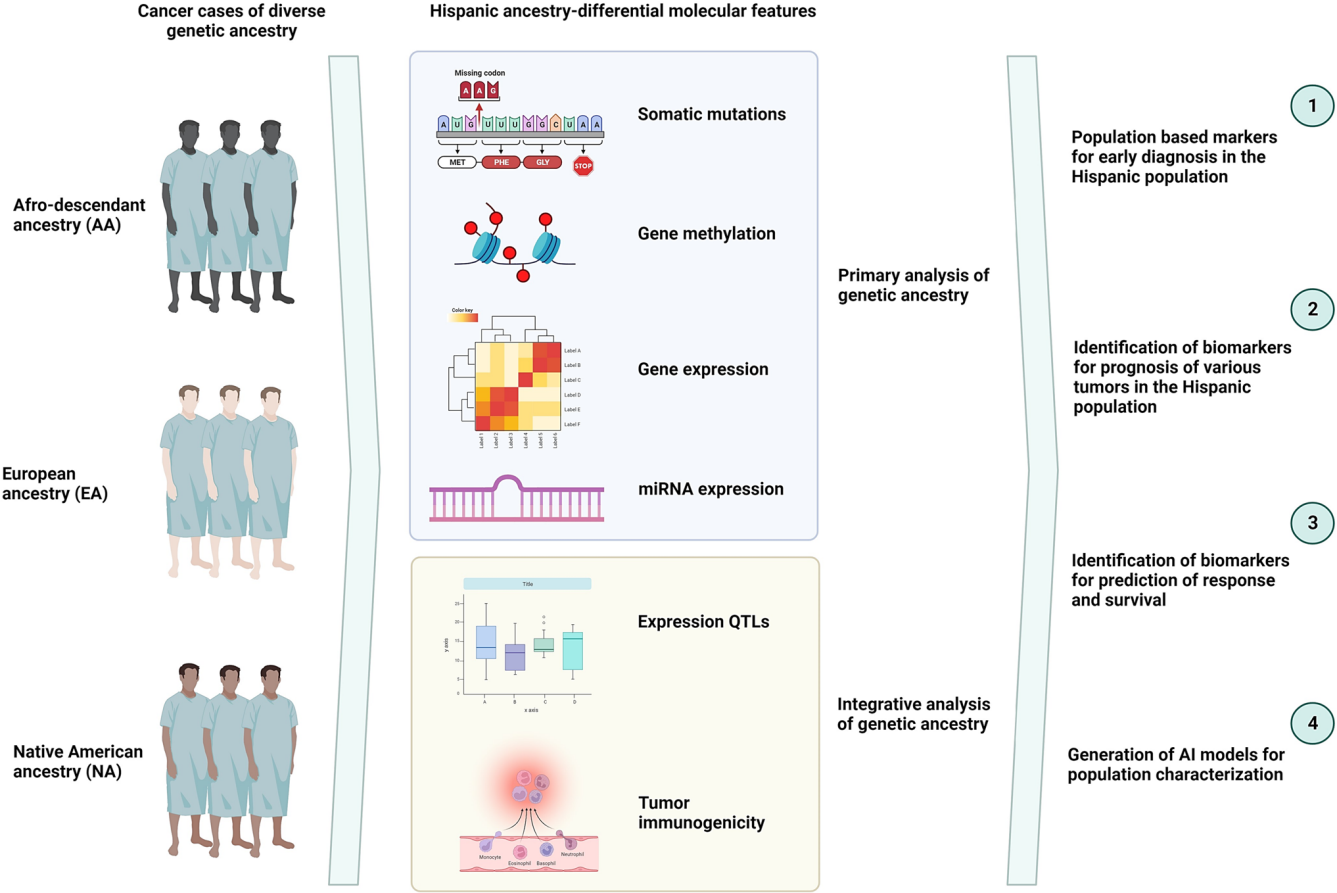
Ancestry-related effects that potentially explain cancer etiology and treatment potentials. Description of diverse ancestries and how different component analyses such as mutational profiling, epigenotype regulation or gene expression define specific subpopulations. Additionally, certain ancestries could relate to divergent QTL expression or immunogenicity. All in all, these differences could alter early diagnoses, the discovery of novel biomarkers for population identification as well as additional population specific therapeutic targets. *QTL* quantitative trait locus

Among Latin Americans, factors contributing to cancer risk partly stem from notable variations in the prevalence of established cancer risk factors within this population. These factors encompass elements such as smoking, inadequate diet quality, and lack of physical activity. Furthermore, restricted access to healthcare and financial limitations are associated with decreased rates of cancer screening [19–21]. Latin Americans often receive diagnoses at later stages for various prevalent cancers, resulting in elevated mortality rates, particularly in the gastrointestinal tract, the biliary tract, the breasts and cervix. This trend could be ascribed to factors such as limited healthcare access, absence of timely detection, and potentially biological influences. Additionally, there has been a noted rise in early-onset disease among Latin Americans in recent years [22]. Significantly, the patterns of cancer occurrence and death rates exhibit extensive variations among diverse subpopulations within Latin America, largely attributed to the differing proportions of the three primary ancestral groups. These proportions might impact the dispersal of cancer susceptibility genes or act as indicators for other closely associated factors such as socioeconomic status, cultural practices, and lifestyle choices [23, 24]. These elements, either collaboratively or individually, could play a role in influencing cancer susceptibility and survival rates. This overview synthesizes existing insights on the genetic heritage pertaining to specific cancers (including breast, lung, colorectal, prostate, gastric, and biliary tract/hepatocellular carcinoma), delving into its implications for epidemiology, biology, and survival. Figure 1 illustrates the effects associated with ancestry that might offer insights into cancer origins and treatment responses among Latin American populations.

## Breast cancer

Breast cancer continues to pose a significant global health concern, with more than 2 million new cases and 600,000 fatalities reported in 2020 [25]. In the context of Latin America, the most prevalent form of neoplasm among women is breast cancer, with Bolivia being the sole exception to this trend [26]. Since the 1990s, global breast cancer mortality rates have been on a steady rise, reaching their peak in Latin America. The region exhibits a particularly high mortality-to-incidence ratio of 0.59, which is twice as high as that reported for the United States [27]. This alarming trend can be associated with a negative correlation stemming from the fact that 60% of breast cancer cases in high-income countries are detected at the earliest stages. In contrast, Brazil and Mexico report early diagnoses in only 20 and 10% of cases, respectively. Notably, Latin America (LATAM) has witnessed a rise in breast cancer mortality rates, particularly in countries such as Brazil, Colombia, Mexico, and Ecuador. Conversely, decreasing trends have been noted in other regions [28].

Breast cancer is characterized by its heterogeneity, encompassing various biological subtypes that hold prognostic significance [29, 30]. In routine clinical practice regarding breast cancer, the subtypes are commonly discerned through immunohistochemical markers, such as the estrogen receptor (ER), progesterone receptor (PR), and human epidermal growth factor receptor 2 (HER2) [31]. Studies conducted across populations and specialized centers consistently demonstrate that women in Latin America face a 20–40% greater likelihood of developing specific subtypes of breast cancer, including ER−/PR−/HER2+ and triple-negative breast cancer (TNBC), when compared to Non-Hispanic White (NHW) women [32].

The association between the risk of breast cancer and genetic ancestry among Latin Americans shows specificity towards tumor subtypes. A study that used data from the San Francisco Bay Area Breast Cancer Study (SFBCS) reported that women with a greater proportion of ancestry from Indigenous America, or conversely, lesser European ancestry, exhibited a decreased risk of breast cancer. The determination of genetic ancestry involved a panel of approximately 100 ancestry informative markers, which did not reveal significant disparities in the proportion of Indigenous American or European genetic ancestry based on estrogen receptor (ER) status. However, a significant discovery was that increased European ancestry remained linked with a heightened overall risk of breast cancer, even after accounting for established risk factors (OR = 1.39; 95% CI 1.06–2.11; *P* = 0.013) [33]. Another supplementary investigation was then carried out with the same data, utilized genome-wide genotype data to estimate ancestry and corroborated the earlier findings [34]. Moreover, a follow-up investigation, incorporating cases from the Kaiser Permanente Pathways Cohort, yielded similar findings. It found no notable correlation between the status of estrogen receptor (ER)/progesterone receptor (PR) or human epidermal growth factor receptor 2 (HER2) and the proportions of ancestries from Indigenous American or European origins [35].

In a recent study by Marker et al., genome-wide genotype data from 1312 patients were analyzed as part of the Peruvian Genetics and Genomics of Breast Cancer Study (PEGEN-BC/Columbus Consortium). This was followed by validation using 616 samples from two more LATAM countries [36]. Globally, there was variation in the average NA ancestry across breast cancer subtypes. Notably, in the multivariate analysis, a 1.2-fold increase in the odds of having a HER2+ tumor was observed for every 10% rise in NA ancestry proportion (95% CI 1.07–1.35; *P* = 0.001). Furthermore, this association between HER2 status and NA ancestry was independently verified in samples from Mexico and Colombia. This suggests that the elevated prevalence of HER2+ tumors among Latin Americans could be influenced by population-specific genetic variants affecting HER2 expression [36] . Furthermore, Zavala et al. explored the correlation between rs140068132 and other polymorphisms in the 6q25 region, examining subtype-specific breast cancer risk in Latin Americans with high Native American (NA) ancestry. Their findings revealed that rs140068132 is associated to a decreased risk of breast cancer in Peruvian adults, providing greater defense against cases with negative hormone receptor (HR−) and HER2+ [37].

Serrano-Gomez et al. examined breast tumor samples from 232 Colombian women, providing insights into average proportions of Native American (NA) ancestry for luminal, HER2-enriched, and triple-negative breast cancer (TNBC) tumors. By utilizing estimates derived from a panel of 80 ancestry informative markers along with immunohistochemistry, they observed that NA ancestry was present in 39, 35, and 37% of luminal tumors, HER2-enriched tumors, and TNBC, respectively. Importantly, there were no statistically significant differences in ancestry observed between these subtypes. However, a sub-analysis considering Colombian region, age at diagnosis, grade, and risk of recurrence revealed significant differences based on intrinsic subtypes [38]. Serrano-Gomez et al. conducted a subsequent study involving a whole-transcriptome RNA-seq analysis in 42 luminal tumors (21 Luminal A and 21 Luminal B) from Colombian women. The analysis categorized genetic ancestry based on luminal subtype and the proportion of European and Native American (NA) ancestry. This examination revealed the potential modulation of five genes influenced by genetic ancestry: HER2, GRB7, GSDMB, MIEN1, and ONECUT2. The replication set confirmed a statistically significant association (*P* = 0.02) between NA ancestry and HER2 expression [39].

Across countries and regions in Latin America, the average African genetic ancestry among Latin Americans/Latinas displays significant variability. In samples from Chile, Argentina, and Mexico, the average African genetic ancestry stands at 5% or less, while in Brazil, Cuba, or Puerto Rico, it reaches 10% or higher [40, 41]. Serrano-Gomez et al. observed a connection between African ancestry and estrogen receptor (ER) status in their study, highlighting that ER−cases exhibited a higher average African ancestry compared to ER+ cases (*P* = 0.02) [38]. Although the SFBCS study did not identify a statistically significant association between African ancestry proportion and ER status, this lack of significance was anticipated due to the relatively small sample size and limited representation of African ancestry in the study [33]. In the PEGEN-BC study, the reported average African ancestry proportion was 4%. Variations in this component among tumor subtypes were insignificant, with proportions ranging between 3 and 5% [36].

Focusing on DNA repair capacity (DRC) as a recognized breast cancer risk factor, several studies have investigated gene expression profiles in Latin American women with breast cancer [42]. In a study conducted by Ramos et al. [43] which compared 33 patients with breast cancer as well as 47 healthy controls from Puerto Rico, a study identified low DNA repair capacity (DRC) as a risk factor for breast cancer among Latin Americans. The results revealed that with every 1% decrease in DRC, there was a corresponding 22% rise in breast cancer risk. Building upon this research, Matta et al. investigated DRC in 824 women (285 breast cancer patients and 539 controls), finding that breast cancer patients displayed diminished levels of DRC [44]. Additionally, within the same research cohort, microarray analyses were conducted to explore the expression patterns of DNA repair genes among Puerto Rican women with breast cancer. This investigation unveiled 21 genes that exhibited differential expression between breast cancer patients and controls. Among these genes were CHEK2, EME1 (MMS4L), ERCC3 (XPB), FANCM, H2AFX (H2AX), HMGB1, HUS1, MBD4, NEIL3, PCNA, RAD1, RAD23B, RAD51, RAD54B, RDM1 (RAD52B), SHFM1 (DSS1), TP1, UBE2N (UBC13), and XRCC5 (Ku80). Furthermore, an analysis of DNA repair capacity (DRC) using the HCR test revealed three genes—RAD51, FANCB, and FANCA—that displayed a positive association with DRC levels [44, 45].

**Fig. 2:**
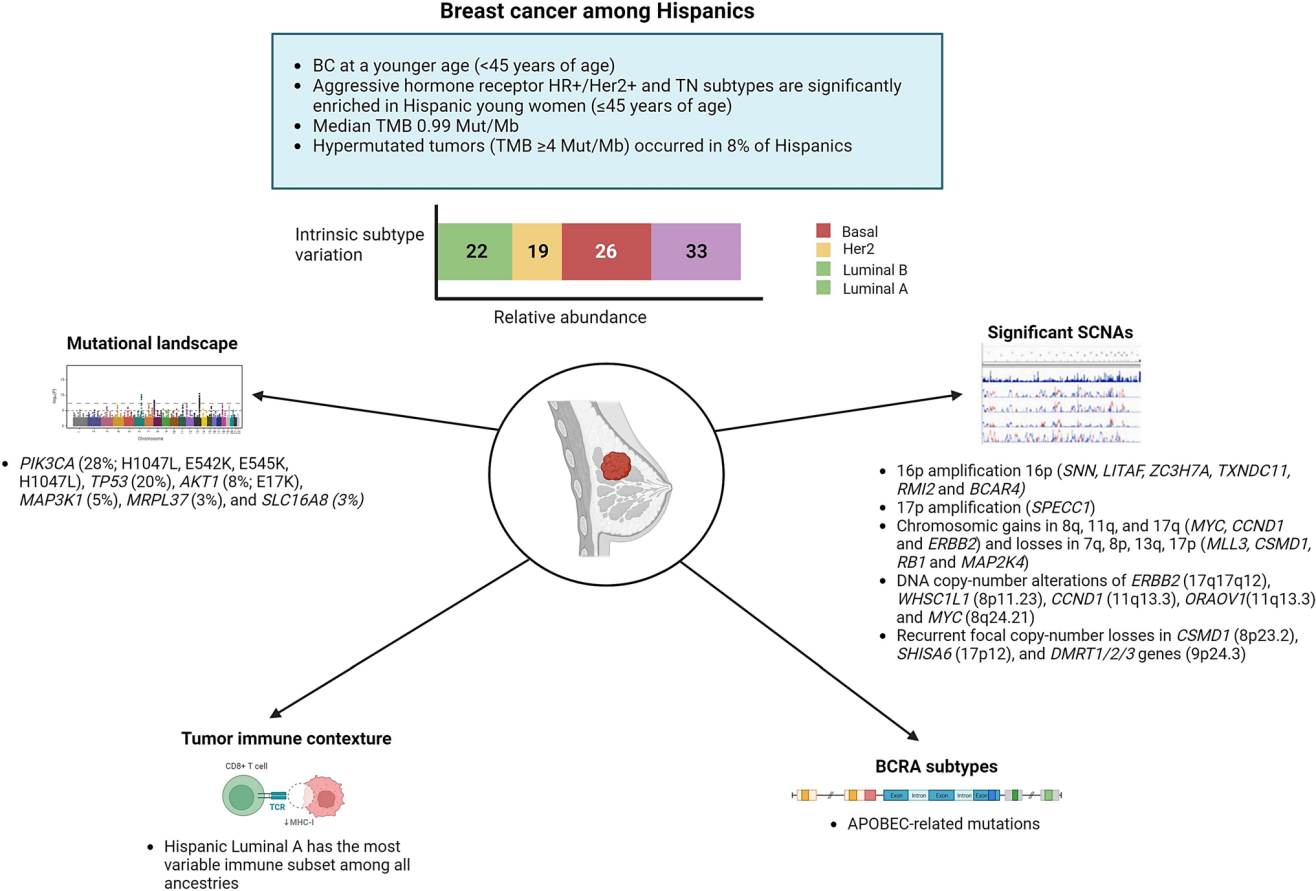
Biological characteristics of breast cancer among Hispanics and their association with NA ancestry

Describing the most extensive genomic analysis of breast cancer among patients with Hispanic-Mexican ancestry in Mexico, Romero-Cordoba et al. provided recent insights [46]. The authors conducted a comprehensive comparison of multi-omics profiles between the Latin American cohort and publicly available data from other ancestries, notably Caucasian, Asian, African, and Afro-American women, to delineate its intricate biological portrait. Their findings revealed that 78% of all tumors harbored at least one driver point mutation, with an average of 2.65 driver mutations, aligning with the rates observed in other ancestries. Additionally, they identified somatic DNA copy-number alterations (SCNA) that were previously undocumented, including the amplification of the region 16p. This region encompasses genes such as SNN, LITAF, ZC3H7A, TXNDC11, RMI2, and the oncogene BCAR4, which have been implicated in endocrine resistance in human breast cancer cells. Furthermore, they observed 17p amplification, where the SPECC1 gene is located [46]. Hispanic-Mexican women also exhibit well-recognized somatic copy number alterations (SCNA) in breast cancer. Notably, gains are observed in chromosomal regions 8q, 11q, and 17q, which house oncogenes like MYC, CCND1, and HER2. Conversely, losses are detected in chromosomes 7q, 8p, 13q, and 17p, which encompass genes such as MLL3, CSMD1, RB1, and MAP2K4. Among the significantly mutated cancer genes identified in over 5% of the cohort are PIK3CA (28%), TP53 (20%), AKT (8%), and MAP3K1 (5%). However, mutations in CDH1 occur at a much lower frequency (2%), while AKT1 mutations are more prevalent (8%) in Hispanic-Mexican women under evaluation. Noteworthy is the Glu17Lys (E17K) mutation within the PHb domain of AKT1, present in 8% of those harboring AKT1 mutations, particularly prominent in HR+ tumors. Additionally, potentially novel mutated genes found in Hispanic-Mexican tumors, exhibiting significant mutation prevalence not previously reported in other datasets, include MRPL37 and SLC16A8 [46]. Figure 2 summarizes the most significant lung cancer findings associated with Latin American ancestry.

Recent research proposes that individuals with approximately 25% African ancestry in Latin America exhibit a heightened prevalence of BRCA pathogenic variants (PVs) [47]. On the contrary, some studies exhibited a reduced output among Latin American immigrants in the US [48].

Reported frequencies of BRCA pathogenic variants (PVs) among Latin American cancer patients range from 1.2 to 16% [49–52] Among young breast or ovarian cancer patients in Mexico, the prevalence ranges from 15 to 28%, regardless of family history of breast cancer [53, 54]. The prevalence and impact of BRCA pathogenic variants (PVs) on the total cancer burden in Latin American populations remain poorly understood. Several studies have investigated the epidemiology of familial breast cancer syndromes in Brazil [55–64]. From 28 centers across 11 Brazilian States, Palmero et al. documented 649 pathogenic/likely pathogenic variants [55]. Small deletions and single nucleotide variants (SNVs) predicted to result in frameshift and non-sense changes in the protein sequence were the most common types of pathogenic variants identified in both BRCA1 and BRCA2 genes. Synonymous pathogenic variants were rare, with only eleven detected in each gene, notably BRCA1 c.4185G > A and BRCA2 c.9117G > A. Large genomic rearrangements (LGRs) comprised 4.9% of the cases, with BRCA2 c.156_157insAlu accounting for 34.3% of all LGR cases.

Among BRCA1 alterations, the nine most common mutations constituted 50.3% of all occurrences, with the European founder mutation c.5266dupC being predominant, accounting for 20.2% of all variants within this gene. Conversely, the mutational spectrum of BRCA2 was more diverse, with non-recurring mutations prevailing at 35.1%, alongside a higher frequency of novel variants. While the most prevalent mutation, BRCA1 c.5266dupC, was observed across all geographical regions, certain recurrent BRCA1 mutations (found in three or more individuals) were distinctive to specific Brazilian states. Variants like c.188T > A, c.2405_2406delTG, c.3916_3917delTT, c.689_692delAGAC, c.4287C > A, and c.5123C > A were solely reported among cases from the Southeastern region (State of São Paulo). Furthermore, mutations such as c.1039_1040delCT and c.1039delC were exclusive to the Northern region (State of Pará), while c.3598C > T and c.5177_5180delGAAA were solely found in carriers of pathogenic mutations from the Southern Region (State of Rio Grande do Sul). No similar regional patterns were observed among recurrent BRCA2 mutations [55]. Regarding ancestry, average proportions were as follows: 70.6% European, 14.5% African, 8.0% Native American, and 6.8% East Asian [62].

Recently, the Hispanic Clinical Cancer Genomics Community Research Network conducted a comprehensive analysis of germline alterations in LATAM. This large-scale study integrated a Hispanic Mutation Panel (HISPANEL) on MassARRAY, semiconductor sequencing, and copy number variant (CNV) detection [65]. In total, 1627 participants were included, with 95.2% diagnosed with cancer. Among them, 236 (14.5%) harbored BRCA pathogenic variants (PVs), with 160 attributed to BRCA1 (31% CNVs) and 76 to BRCA2. The frequency of BRCA PVs varied across countries, with rates of 26% for Brazil, 9% for Colombia, 13% for Peru, and 17% for Mexico. Recurrent PVs, observed three or more times, accounted for 42.8% of all PVs. Additionally, 14% of unique PVs lacked entries in ClinVar, and 57% of unique variants of unknown clinical significance (VUS) had no known ClinVar entry [65].

## Lung cancer

On a global scale, lung cancer emerges as the most prevalent malignancy and the leading cause of cancer-related fatalities. In 2022, approximately 2.2 million new diagnoses were reported, constituting 11.6% of the cancer incidence burden [66]. The Global Burden of Disease Study 2020 underscores the substantial healthcare burden linked with lung cancer worldwide. Notably, the 5-year survival rate for lung cancer stands at a mere 17.8%, considerably lower than that of other prominent cancers [67]. With an alarming 83% fatality rate [68], the geographical mortality patterns closely mirror the incidence, making lung cancer a significant public health concern. In LATAM, lung cancer exacts an especially heavy toll, surpassing other malignancies in terms of mortality. According to the International Agency for Research on Cancer, over 80,000 individuals succumbed to lung cancer in the LATAM region in 2022. This signifies a loss of over 30,000 lives compared to the next most lethal cancer, constituting approximately 14% of all deaths related to neoplasm [69].

Across Latin American (LATAM) countries, analyses of EGFR mutation frequencies in adenocarcinomas reveal varying rates. Approximately 15% are reported in Argentina, 20–25% in Brazil, 25–35% in Mexico, Costa Rica, and Colombia, and 40–50% in Peru [70]. Peru, with a predominantly NA descent population influenced by migrations from East Asia, particularly China and Japan, stands out. Conversely, Brazil, Mexico, Costa Rica, and Colombia have mixed populations, while Argentina and Uruguay, characterized by a strong history of European immigration, demonstrate the lowest frequencies of EGFR mutations in LATAM [71]. Implying a possible link between the frequency of somatic mutations in EGFR in lung cancer and the genetic ancestry of populations, these findings underscore the need for deeper investigation. It is crucial to grasp the panorama of somatic cancer mutations in lung cancers originating from Latin America and assess the influence of germline ancestry on these somatic alterations. In their study, Carrot-Zhang et al. performed genomic analysis on 601 lung cancer cases from Mexico and 552 from Colombia, encompassing 499 self-reported non-smokers [72]. In the study, oncogenic mutations in EGFR, KRAS, BRAF, ERBB2, MET, or fusions in ALK, ROS1, or RET were identified in 552 (48%) samples. Moreover, a broader set of known lung cancer driver genes, including TP53, STK11, KEAP1, SMARCA4, SETD2, MYC, and MDM2, harbored at least one detectable alteration in 68% of all samples [72]. In the tested lung cancer samples from Mexican patients, the mutation frequencies of EGFR and KRAS were 30 and 10%, respectively, while in Colombian patients, these frequencies were 23 and 13%. Analysis of somatic copy number alterations (SCNA) revealed that 9% of cases exhibited high-level amplifications in MYC and 2% in MDM2. When evaluating the association between ancestry and mutations, after adjusting for sample-specific tumor mutation burden (TMB), each gene showed distinct patterns. Specifically, Native American (NA) ancestry was positively correlated with mutations in EGFR (*P* = 0.005) and inversely correlated with mutations in KRAS (*P* = 0.00001) and STK11. These findings align with previous studies focusing on Asian patients [72, 73]. In patients who have never smoked, the TMB and NA ancestry association was more robust in EGFR-mutant (*P* = 0.002) than in EGFR-wild type (*P* = 0.038). Moreover, the joint model (TMB–NA ancestry + EGFR + EGFR * NA ancestry) revealed a significant association between EGFR and NA ancestry interaction and TMB (*P* = 0.04), indicating that the relationship between TMB and NA ancestry differs between EGFR-mutant and EGFR-wild type samples. However, the effect size of ancestry on KRAS was not modified by the interaction of smoking signature and NA ancestry (*P* = 0.34), and mutations of lung cancer oncogenes were not associated with gender and APOBEC signatures [72]. These findings follow a population pattern confirmed by other studies developed in Brazil and the Latin American population from the US [74–76]. It was also found that disruptive/truncating mutations of TP53 are related to a worse prognosis in lung adenocarcinomas, especially in young and Afro-descendant patients [77].

**Fig. 3:**
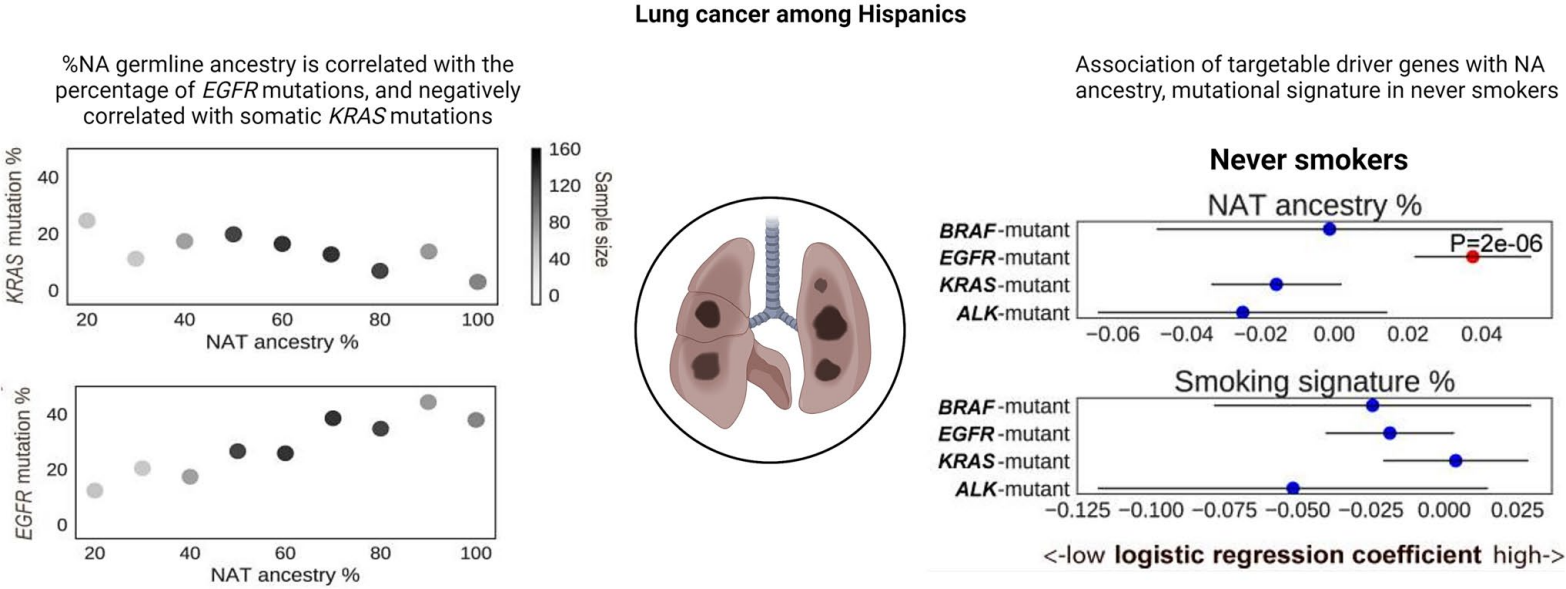
Targetable lung cancer driver genes associated with genetic ancestry among Hispanics (Modified from Carrot-Zhang J, Soca-Chafre G, Patterson N, Thorner AR, Nag A, Watson J, Genovese G, Rodriguez J, et al. Genetic Ancestry Contributes to Somatic Mutations in Lung Cancers from Admixed Latin American Populations. Cancer Discov. 2021 Mar;11(3):591–598)

Reported by Cardona et al., squamous cell lung cancer’s molecular profile reveals a significant prevalence of inactivating mutations in TP53 (61.5%), PIK3CA (34.6%), MLL2 (34.6%), KEAP1 (38.4%), and NOTCH1 (26.9%). PD-L1 expression varied from negative, 1, 2–49, and ≥50% in 23.1, 38.5, 26.9, and 11.5% of cases, respectively [78]. Figure 3 shows the percentage of NA germline ancestry, and it´s correlation with somatic EGFR mutations and anticorrelation with KRAS mutations.

## Colorectal cancer

Colorectal cancer (CRC) distribution exhibits global variations influenced by geographical region and age group. Developed countries show higher incidence rates, whereas over the past 25 years, developing countries have had a notable surge in colorectal cancer (CRC) incidence [79]. This surge is attributed to factors such as demographic transition, expanded access to healthcare, screening initiatives, improved socioeconomic indicators, and lifestyle changes, leading to increased exposure to risk factors [80]. However, the specific factors influencing CRC trends in Latin American countries remain to be fully elucidated. Between 1990 and 2019, there was a 20.6% rise in the CRC-adjusted mortality rate across Latin America, showcasing an average annual percentage change (APC) of 0.11% per year. Significant heterogeneity among countries within the region was observed during this period [81].

Seagle et al. recently conducted a thorough ancestry analysis employing Multigene Panel Testing (MPT) to discern racial and ethnic disparities in germline PVs among early-onset CRC patients. The study unveiled that 14% of Latin American patients harbored some germline PVs, with an added risk observed for alterations in the APC gene, including the mutation p.I1307K (OR 2.67; 95% CI 1.30–5.49; *P* = 0.007) and MLH1 (OR 8.69; 95% CI 2.68–28.20; *P* = 0.0003) compared to non-Hispanic whites. Moreover, significant heterogeneity was noted among the countries in the region [82]. The spectrum of mismatch repair gene mutations in 397 individuals with Lynch syndrome from Latin America was reported by Sunga et al. [83]. In this cohort spanning multiple centers, 79 sequence alterations in MMR genes were detected among 77 probands from unrelated families. Of these, 71 alterations were classified as pathogenic or likely pathogenic, with nine categorized as VUS. The predominant mutations were frameshift (29.8%) and nonsense (26.3%), followed by splice site (21.1%), large exonic deletions (12.3%), and missense mutations (10.5%). Among all genomic alterations, variants such as MLH1 c.350C > T, c.2041G > A, c.332C > T, and c.676C > T, and MSH2 c.1216C > T, deletion of exons 4–8, and deletion of exon 8, as well as PMS2 deletion of exon 14, had been previously documented in Spain [84]. In one Mexican family, the MSH2 deletion of exons 4–8 was observed, previously identified as a Spanish founding mutation [85]. The presence of multiple mutations in this cohort, previously documented in Spain, aligns with established migration routes of Europeans to Mexico and Central America.

Examining the link between genetic ancestry and colorectal neoplasms in Colombia, Hernandez-Suarez et al. scrutinized 190 adenocarcinomas, 113 sporadic adenomas, and 243 age- and sex-matched controls. The researchers utilized the STRUCTURE software, taking into account three separate population origins determined by allele frequencies [86]. In the results, adenomas (mean fraction 0.13, 95% CI 0.11–0.15) and cancer cases (mean fraction 0.14 95% CI 0.12–0.16) exhibited a greater average African ancestry fraction compared to controls (mean fraction 0.11 95% CI 0.10–0.12) [86]. Additionally, upon conducting conditional logistic regression analysis and accounting for established risk factors, it was observed that a 10% rise in African ancestry displayed a favorable correlation with both colorectal adenoma (OR 1.12 95% CI 0.97–1.30) and adenocarcinoma (OR 1.19 95% CI 1.05–1.35) [86]. In an effort to grasp the molecular pathways possibly underlying the observed health disparities in colorectal cancer (CRC) among Latin Americans, Schmit et al. delved into an analysis of somatic molecular markers in 488 Puerto Rican patients. Their findings showcased that the majority of tumors were microsatellite stable (98.4%), exhibited a low level of CpG island methylator phenotype (CIMP-low) (92.1%), and possessed wild-type KRAS (68.8%) and BRAF (90.8%) genes [87]. Among Latin Americans in the United States, colorectal cancer (CRC) tumors exhibit distinctive characteristics compared to other ethnic or racial groups. These include a lower incidence of microsatellite instability (MSI), reduced prevalence of CIMP-high tumors, and decreased mutation rates for critical CRC driver genes. Furthermore, a separate study focused on understanding the genetic predisposition to CRC among Puerto Ricans. This investigation delved into the connection between ancestry and heightened cancer risk among 425 controls, 99 adenomas, and 414 CRC cases. The results unveiled a trend of increased risk with rising levels of European ancestry. Conversely, Puerto Rican individuals with above-average levels of West African ancestry faced a heightened risk of presenting with CRC in the distal colon, characterized by moderate or low differentiation and accompanied by BRAF mutations [88].

Recent research indicates that the mutation profile of colorectal cancer (CRC) patients in Brazil closely resembles that of other populations [89, 90]. In a series of 91 Brazilian colorectal cancer (CRC) patients, Dos Santos et al. explored the mutation profile of 150 cancer-related genes using next-generation sequencing (NGS) and microsatellite instability (MSI), while examining their connection with genetic ancestry. Driver mutations were detected in APC (71.4%), TP53 (56.0%), KRAS (52.7%), PIK3CA (15.4%), and FBXW7 (10.9%) [89]. In the study, mutations in genes of the MAPK/ERK, PIK3/AKT, NOTCH, and receptor tyrosine kinase signaling pathways were observed in 68.0, 23.1, 16.5, and 15.3% of patients, respectively. Additionally, MSI was detected in 13.3% of tumors, with a majority being proximal (52.4%, *P* < 0.001) and exhibiting a high TMB. The predominant genetic ancestry was European (83.1%), followed by Native American (4.1%), Asian (3.4%), and African (3.2%). Notably, NF1 and BRAF mutations were associated with African ancestry, while TP53 and PIK3CA mutations showed an inverse correlation with Native American ancestry [89]. Oliveira Durães et al. recently elucidated the impact of genetic ancestry in 1002 Brazilian CRC patients from Barretos Cancer Hospital [90]. The analysis revealed a strong admixture composition in 934 cases, with a predominant proportion of European ancestry (74.2%), followed by African (12.7%), Native American (NA) (6.6%), and Asian (6.5%) [90]. Their investigation extended to exploring the correlation of patients’ clinicopathological characteristics with genetic ancestry, They discovered significant associations between the African component and younger age at diagnosis (*P* = 0.013), origin from the Brazilian region (*P* < 0.001), and disease recurrence (*P* = 0.034). Regarding the European component, significant associations were found with the region of origin (*P * < 0.001), higher histological grade (*P* = 0.040), and the presence of synchronous tumors (*P* = 0.012). As for the Native American (NA) ancestry component, a notable association with the mucinous histological subtype (*P* = 0.033) emerged [90].

## Prostate cancer

Prostate cancer ranks as one of the most prevalent diseases globally among men, with GLOBOCAN estimates reporting nearly 1.3 million new cases and 360,000 prostate cancer-related deaths in 2020 [66]. In Latin America (LATAM), it stands as the third most common tumor (following lung and breast cancers), and notably, it’s the most frequent cancer among men, carrying the highest mortality rate of all cancers. Projections for 2022 suggest approximately 170,000 new cases and 55,000 regional deaths attributed to prostate cancer [91]. Across most countries in LATAM, including Brazil, Ecuador, Colombia, and Costa Rica, both the incidence and mortality rates of prostate cancer continue to climb. This upward trend is influenced by evolving risk factors, increasing longevity in the population, and challenges in accessing adequate local or systemic treatments [92].

From the Million Veteran Program data and other independent studies, Chen et al. constructed a polygenic risk score (PRS) spanning multiple ancestries, effectively stratifying prostate cancer risk among diverse populations [93]. The evaluation encompassed 31,925 cases and 490,507 controls, including 1082 cases and 20,601 controls from Latin American backgrounds. Comparing men within the top decile (90–100% PRS) to those in the 40–60% PRS range, the odds ratio (OR) for prostate cancer was 3, eightfold higher in European ancestry individuals (95%CI 3.62–3.96), 2, eightfold higher in African ancestry individuals (95% CI 2.59–3.03), and 3, twofold higher in Latin American individuals (95% CI 2.64–3.92) [93]. In their study, Du et al. conducted a genome-wide association study (GWAS) of prostate cancer, analyzing 2820 cases and 5293 controls from Latin America. Their aim was to uncover new risk loci and develop a genetic admixture mapping method to pinpoint risk alleles linked with local ancestry [94]. Significant associations across the genome were observed with 84 variants, all concentrated in the established prostate cancer risk regions at 8q24 and 10q11.22 (MSMB gene). The study also identified a significant genome-wide association with local African ancestry at 8q24 through admixture mapping. Moreover, among the 162 established prostate cancer risk variants common among Latin American men, 83.3% showed effects consistent with the disease. A polygenic risk model incorporating these known risk variants revealed that men in the top 10% had a 3.19-fold (95% CI 2.65–3.84) increased prostate cancer risk compared to those with average risk (25th–75th percentile of the PRS distribution) [94]. Additionally, another GWAS examining prostate cancer in Kaiser Permanente health plan members (comprising 7783 cases and 38,595 controls, with 80.3% non-Hispanic white, 4.9% African American, 7.0% East Asian, and 7.8% Latino) uncovered a novel independent risk indel, rs4646284, at the locus 6q25.3 that was previously identified [95]. Comparing the highest to lowest risk score deciles across the 6q25.3 locus, rs4646284 showed the strongest association with the expression of SLC22A1 and SLC22A3 genes. The odds ratio (OR) was 6.22 for non-Hispanic whites, 5.82 for Latin Americans, 3.77 for African Americans, and 3.38 for East Asians [95].

With targeted next-generation sequencing under the GENIE 11th model, Arenas-Gallo et al. evaluated 1412 primary and 818 metastatic prostate adenocarcinomas, including Latin American men [96]. The study determined that TMPRSS2 and ERG gene alterations in primary tumors were more common among Latin Americans (51.28%; OR 0.44 95% CI 0.27–0.72). On the other hand, in metastatic tumors, KRAS and CCNE1 alterations were less prevalent in non-Hispanic White men, and no significant differences were found in actionable alterations and androgen receptor mutations between the groups [96]. Developing a retrospective analysis, The Hispanic Americans Prostate Cancer Comprehensive Genomic Profiling Study (THAPCA-GPS) examined 190 patients with metastatic prostate adenocarcinoma. Among them, 24.2% were of Latin American origin. The study aimed to determine the status of Homologous recombination repair (HRR) in somatic tissue, liquid, and germline-test [97]. Compared to Non-Hispanic Whites, Latin Americans exhibited a higher proportion of TMB-High > 10 (30 vs. 3.6%, *P* = 0.02), PD-L1 CPS > 5 (9.4 vs. 0%, *P* = 0.03), and TMPRSS2-ERG fusion (37.5 vs. 7.8%, *P* = 0.0009), as revealed by the study [97]. In a retrospective study by Shaya et al., the rate of pathogenic/likely pathogenic (PLP) germline alterations in Latin American men was assessed across 25 genes commonly linked to prostate cancer. The frequency was compared with a cohort of Non-Hispanic Whites [98]; Identified were 508 Latin Americans and 12,542 Non-Hispanic whites, exhibiting alteration rates of 7.1 and 9.7% for the PLP, respectively (*P* = 0.058). Notably, the Latin American cohort showed a notably higher rate of VUS. The four most commonly detected genes with PLP alterations in both cohorts were ATM, BRCA1, BRCA2, and CHEK2. Interestingly, only the rate of CHEK2 alterations differed significantly between the cohorts of Non-Hispanic whites [98].

## Gastric cancer

By 2020, reports indicated approximately 1.1 million new cases of gastric cancer and 770,000 associated deaths. On average, incidence rates were twofold higher in males than in females, reaching 15.8 and 7.0 per 100,000, respectively. These rates varied across countries, with the highest recorded incidences observed in Eastern Asia and Latin America. Predictions suggest that by 2040, the annual burden of gastric cancer will escalate to approximately 1.8 million new cases and 1.3 million deaths [99]. In comparison to individuals of other ethnicities and races, gastric cancer patients in Latin America manifest distinct clinicopathologic features. Latin Americans in the US, on the other hand, experience double the incidence and mortality rates from gastric cancer compared to non-Hispanic Whites [100]. Furthermore, Latin American patients with gastric cancer frequently exhibit a higher proportion of diffuse-type cancers (DGC), are diagnosed at a younger age, and present with more advanced-stage disease [101, 102]. Ethnicity-associated disparities in tumor biology may play a role, alongside environmental exposures and socioeconomic factors, in contributing to the observed clinicopathologic differences.

To address the knowledge gap created by the limited representation of Latin American patients in the Cancer Genome Atlas (TCGA) study, Wang et al. conducted an extensive, integrated genomic analysis of 83 gastric cancer patients from Latin America. This study aimed to fill the void left by the TCGA’s study of gastric adenocarcinoma, which included only five Latin American patients among its 478-patient cohort. Comparative analyses were then conducted using data from Asian and White patients previously published by TCGA [103]. Latin Americans, when compared to Asian (20%) and White (21%) patients, demonstrated a notably higher proportion of genomically stable tumors (GST) (65%, *P* < 0.001). Among Latin Americans, CIN samples exhibited an average of 3.5 Mut/Mb, while GST had 2.0 Mut/Mb. TP53 emerged as the most common recurrent mutation in Latin American gastric cancer samples, mirroring findings from the TCGA. RNASeq analysis revealed 4 cases carrying the CLDN18-ARHGAP fusion, which was the most frequent discovery in GSTs. Wang et al., like with the TCGA data, identified alterations in the 8q24.21 region housing the MYC oncogene and KRAS amplification (12p12.1) in CIN patients. The incidence of PIK3CA mutations, mainly found in EBV-type tumors, was notably absent in the Latin American population. Conversely, Latin American CIN tumors exhibited a lower rate of TP53 mutations (35 vs. 70% for Non-Hispanic Whites) but a higher incidence of APC mutations (29 vs. 10%). The study also identified a lower rate of alterations in RHOA (3 vs. 18%, sum of both CIN and GS) and ARID1A (8 vs. 25%, sum of both CIN and GS) among Latin Americans [103].

**Fig. 4:**
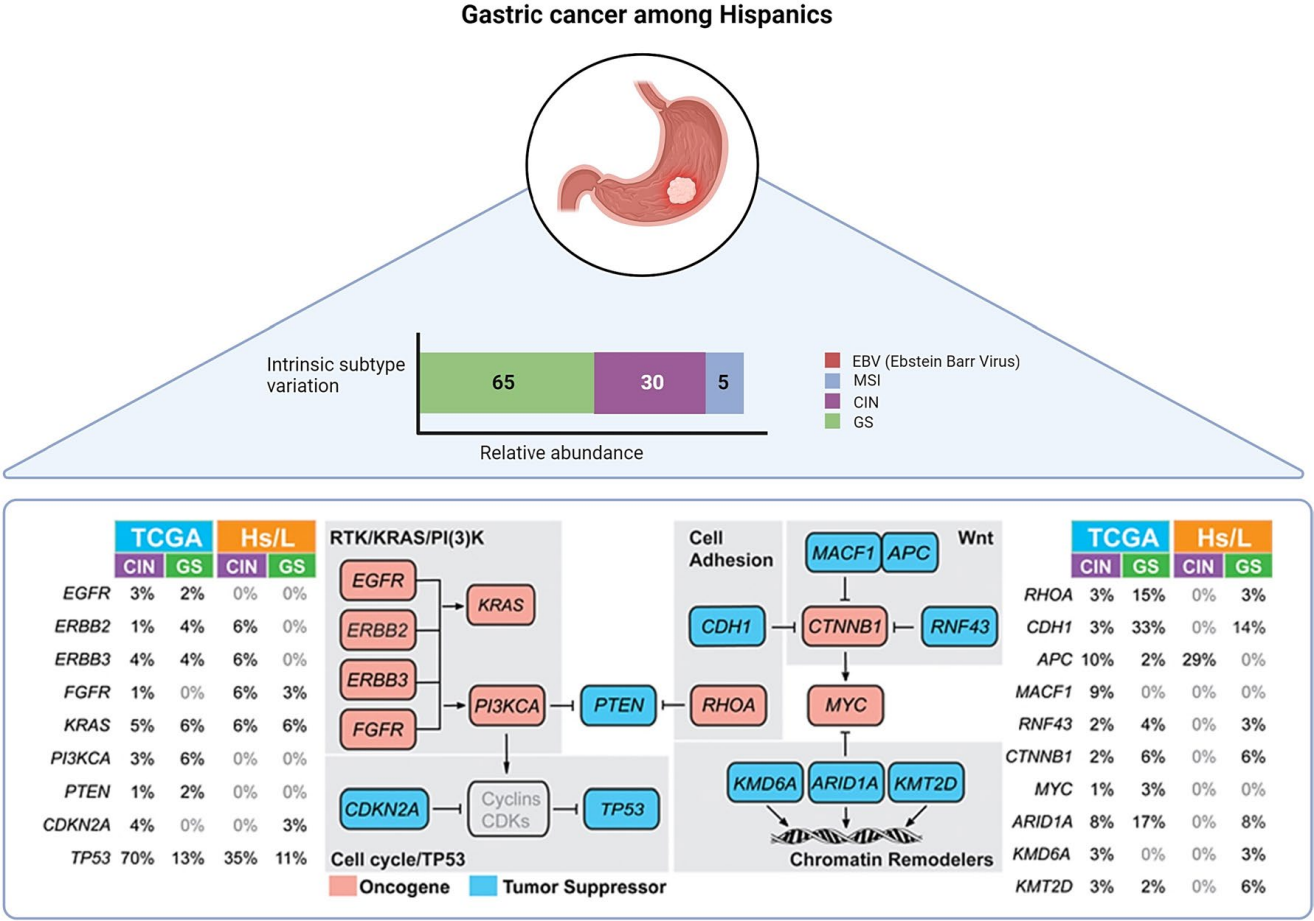
Key genomic features of gastric cancer are identified among Hispanics. Comparison of incidence of somatic alterations in select genes involved in RTK/RAS/PI(3)K signaling, cell cycle, cell adhesion, Wnt signaling, and chromatin remodeling, in the TCGA and Hispanics, stratified by CIN (chromosomal instability) and GS (genomically stable) subtypes (Modified from Wang SC, Yeu Y, Hammer STG, Xiao S, Zhu M, Hong C, Clemenceau JR, Yoon LY, Nassour I, Shen J, Agarwal D, Reznik SI, Mansour JC, Yopp AC, Zhu H, Hwang TH, Porembka MR. Hispanic/Latino Patients with Gastric Adenocarcinoma Have Distinct Molecular Profiles Including a High Rate of Germline CDH1 Variants. Cancer Res. 2020 Jun 1;80(11):2114–2124)

Toal et al. recently carried out an evaluation of gastric intratumoral heterogeneity using multiregional sequencing. Their study encompassed over 700 cancer genes and included 115 tumor biopsies from 32 patients, of whom 29 had Latin American ancestry, relative to the TCGA study [104]. The study findings unveiled that roughly 30% of all mutations were clonal, with 61% of known TCGA gastric cancer drivers showcasing clonal mutations. Additionally, new potential gastric cancer drivers, namely EYS, FAT4, PCDHA1, RAD50, EXO1, RECQL4, and FSIP2, demonstrated multiple clonal mutations. Furthermore, the GST molecular subtype, linked to a worse prognosis, was identified in 48% of Latin American patients, surpassing that of TCGA Asian and White patients by more than 2.3-fold [104]. In microsatellite-stable tumors, mutation signature analyses revealed common DNA repair mutations during both tumor initiation and progression. Additionally, only a third of all tumors exhibited clonal pathogenic mutations in druggable genes. Initiators like tobacco, POLE mutations, and inflammation signatures are likely to contribute to carcinogenesis [104]. Figure 4. Key genomic features of gastric cancer are identified among Latin Americans.

## Hepatocellular and biliary tract and carcinomas

Globally, liver cancer ranks as the fourth leading cause of death, resulting in over 800,000 fatalities each year [105]. Representing about 90% of primary liver cancers, hepatocellular carcinoma is followed by intrahepatic cholangiocarcinoma and other primary liver malignancies. Known underlying causes are associated with approximately 90% of hepatocellular carcinomas, with chronic viral hepatitis, heavy alcohol use, and non-alcoholic fatty liver disease being the most common [105]. Geographically, there is significant variation in the distribution of these etiologies [106, 107]. In East Asia, there is a higher prevalence of chronic viral hepatitis, whereas in Europe, heavy alcohol use is most prominent. Additionally, the regional differences in liver cancer incidence and mortality rates further underscore the varying landscape [106, 107]. Latin Americans currently bear a disproportionate burden of hepatocellular carcinoma (HCC), with an incidence and cancer-related mortality nearly double that of non-Hispanic whites [105].

Developing a study, Das et al. analyzed 31 paired tumor and adjacent non-tumor liver samples from Latin American patients. This aimed to evaluate the genomic characteristics and underlying molecular mechanisms driving tumorigenesis associated with HCC [108]. In Latin American HCC, like in other ethnic groups, the most frequent somatic mutations were found in CTNNB1 and TP53. Conversely, AXIN2 and, to a lesser extent, MTOR mutations appear more commonly observed in Latin Americans than in other ethnic groups analyzed in the TCGA study [109]. Signatures of tobacco and aflatoxin exposure were observed in somatic mutations found in Latin American HCC, diverging from those commonly found in liver cancer patients in other reports. The most frequently altered oncogenic pathways included WNT, TP53, and the cell cycle. Through integrated transcriptomic, proteomic, and metabolomic analyses, significant negative enrichments were identified in gluconeogenesis, the TCA cycle, and glutamate metabolism in Latin American HCC. These findings hint at the presence of molecular mechanisms that are either unique to Latin Americans or differ from those in non-Latin American HCC [108]. Lin et al. reported the frequency of commonly mutated genes in a cohort of Latin American patients with HCC compared to non-Latin Americans. The frequencies for CTNNB1 were 45.5 versus 44.4%, for TERT were 45.5 versus 55.6%, for TP53 were 36.4 versus 55%, and for CNK2A were 18.2% versus 0, respectively [110]. Detected commonly in TP53 mutations at codon 249(R249S), the median allele frequency (AF) percentage stood at 0.81%. The presence of these pathogenic mutations correlated with adverse clinical features, including multifocal disease and higher AFP values (>59 ng/dl), in comparison to other genomic alterations. Other mutations, such as ERBB2, PIK3CA, DNMT3A, GNAS, KDR, RB1, PTEN, were present at lower percentages but exhibited similar frequencies in both Latin American and non-Latin American groups [110].

Annually, approximately 116,000 individuals receive a diagnosis of gallbladder cancer (GBC), with 85,000 succumbing to this aggressive disease globally [111]. Women in low- and middle-income countries primarily bear the brunt of biliary tract malignancy [112]. Until the disease has progressed to an advanced stage, leaving patients with few treatment options, symptoms of gallbladder cancer (GBC) are often absent or unspecific, a condition related to environmental exposures and genetic predisposition [112]. Mainly diagnosed at an advanced stage, GBC exhibits a low 5-year survival rate. Studies indicate rates ranging between 5 and 30%, contingent upon the country of origin of the study population [113]. A wide geographical and ethnic variation is observed in the incidence of GBC [111]. In contrast to high-income regions like Western Europe, the United States, and Australia, which report 2 cases per 100,000 person-years, the Mapuche, the most significant indigenous group in Chile, exhibit the world’s highest incidence of gallbladder cancer (GBC), with ≥20 cases per 100,000 person-years [114]. In observational studies, a robust correlation has been discovered between the individual proportion of Native American (NA) ancestry and gallbladder cancer (GBC) risk. Specifically, for every 1% rise in Mapuche ancestry, there is a corresponding 3.7% increase in GBC mortality [114]. The association observed, however, may stem from other established risk factors for gallbladder cancer (GBC), particularly gallstones and higher body mass index (BMI) [115]. Recent studies have discovered evidence of a causal effect of gallstones on gallbladder cancer (GBC) risk for genetically admixed Chileans, with an odds ratio (OR) of 1.97. This observation follows a relative GBC risk of 4.9 observed in individuals with a history of gallstones [116, 117]. An increased BMI, diabetes, and asthma have also been linked to NA ancestry [117].

## Conclusion

People of different ancestries exhibit varying germline genetics, cancer incidence, outcomes, and molecular characteristics [118–120]. However, the inclusion of Latin American patients in large cohort studies does not exceed 2%, thereby limiting the analysis of molecular epidemiology and ancestry of solid and hematological tumors in this mestizo population to small studies. These smaller studies may potentially fail to identify the magnitude of inter and intra population variance. Despite limitations, molecular characterization of tumors and germline evaluations reveal that population composition sometimes diverges from the expected molecular epidemiology in European or North American individuals. In summary, this comprehensive examination of genetic ancestry in Latin American populations sheds light on its profound implications for health. These implications range from the development of innovative genetic atlases to understanding cancer determinants. The findings underscore the need for nuanced approaches in research and healthcare. Such approaches should consider the intricate interplay of genetic, socio-economic, cultural, and lifestyle factors in determining health outcomes in this diverse and dynamic population.

## Data Availability

The article is a review, it has no original data available to readers.
